# The neural mechanism of aesthetic judgments of dynamic landscapes: an fMRI study

**DOI:** 10.1038/s41598-020-77658-y

**Published:** 2020-11-27

**Authors:** Xueru Zhao, Junjing Wang, Jinhui Li, Guang Luo, Ting Li, Anjan Chatterjee, Wei Zhang, Xianyou He

**Affiliations:** 1 School of Psychology, South China Normal University, Guangzhou, China; 2 Academy of Basic Education Professionals, Beijing Institute of Education, Beijing, China; 3 Key Laboratory of Brain, Cognition and Education Sciences (South China Normal University), Ministry of Education, Guangzhou, China; 4 Center for Studies of Psychological Application, South China Normal University, Guangzhou, China; 5 Guangdong Key Laboratory of Mental Health and Cognitive Science, South China Normal University, Guangzhou, China; 6 Department of Applied Psychology, Guangdong University of Foreign Studies, Guangzhou, China; 7 School of Fine Arts, South China Normal University, Guangzhou, China; 8 SSL Experimental Middle School, Dongguan, China; 9 School of Medicine, University of Pennsylvania, Philadelphia, USA

**Keywords:** Psychology, Human behaviour

## Abstract

Most previous neuroaesthetics research has been limited to considering the aesthetic judgment of static stimuli, with few studies examining the aesthetic judgment of dynamic stimuli. The present study explored the neural mechanisms underlying aesthetic judgment of dynamic landscapes, and compared the neural mechanisms between the aesthetic judgments of dynamic landscapes and static ones. Participants were scanned while they performed aesthetic judgments on dynamic landscapes and matched static ones. The results revealed regions of occipital lobe, frontal lobe, supplementary motor area, cingulate cortex and insula were commonly activated both in the aesthetic judgments of dynamic and static landscapes. Furthermore, compared to static landscapes, stronger activations of middle temporal gyrus (MT/V5), and hippocampus were found in the aesthetic judgments of dynamic landscapes. This study provided neural evidence that visual processing related regions, emotion-related regions were more active when viewing dynamic landscapes than static ones, which also indicated that dynamic stimuli were more beautiful than static ones.

## Introduction

“Loving beauty is part of human nature.” In past centuries, philosophers, thinkers, and psychologists have emphasized the importance of beauty. With the rise of neuroaesthetics, researchers are paying more attention to physiological basis underlying aesthetic experiences.

A number of studies have examined neural mechanisms underlying aesthetic judgments. The aesthetic judgments of representational paintings revealed significant activation in ventral occipital poles, posterior middle temporal gyrus, and precuneus compared with the aesthetic judgments of abstract paintings^1^. It’s worth mentioning that aesthetic experience is not art-specific. Some non-art works, such as geometrical shapes, human faces, will also arouse the viewer’s aesthetic experience. The aesthetic judgment of geometrical shapes significantly activated the frontomedian cortex, frontal gyrus, cingulate cortex, temporal pole, and the temporoparietal junction compared with symmetry judgments^2^. Winston, et al. found that medial prefrontal and paracingulate cortices, posterior OFC, insula, and superior temporal sulcus were significantly activated in the attractiveness judgments versus age judgments^3^.

It is generally believed that regions associated with sensory, cognitive, emotional and reward processing are commonly activated in aestheticjudgments^4–8^. Previous studies report that aesthetic judgments of visual stimuli produce greater activation in the occipital regions associated with visual processing^1,9–12^. Additionally, the frontal cortex, a region involved in cognitive processing, also plays an important part in aesthetic judgments^3,13,14^. Emotional and reward processing areas also undergird aesthetic judgments in a way that adds to general cognitive activities. The emotional and reward experience triggered during aesthetic judgments is reflected in the hippocampus, insula, amygdala, anterior cingulate gyrus and orbitofrontal cortex ^1,9,15–18^.

However, these results are mainly based on using static visual stimuli. People also find dynamic objects beautiful. How does the brain process dynamic visual stimuli when we experience their beauty? Aesthetic judgments of both static stimuli and dynamic stimuli would undoubtedly include sensory, cognitive and emotional processing. But, what is similar or different in the neural mechanism underlying aesthetic judgments of dynamic and static stimuli?

Prior neuroimaging evidence showed that area V5/MT plays an important role in the perception of visual motion, which also indicated that MT may be a special brain area for dynamic stimuli versus static stimuli^19–22^. Patterns of moving dots produced activity in areas V1, V2, the V3 complex (V3, V3A, V3B) and V5^23^. Even static stimuli with a high dynamic content might induce stronger activity in V5/MT^24^. Furthermore, motor cortex areas were also found to be related to dynamism perceived in the painting^25,26^. The source localization analysis of EEG data showed that viewing abstract works characterized by the presence of marked traces of brushstrokes elicited the activation of premotor and motor cortical areas, reward-related orbitofrontal areas, and cognitive categorization-related prefrontal areas^27^. Similarly, the observation of cut canvases of Lucio Fontana is associated with specific cortical motor activation^28^.

Few studies have directly explored the neural mechanism of the aesthetic judgments of dynamic visual stimuli. Calvo-Merino, Jola, Glaser and Haggard investigated the neural correlates of implicit aesthetic responses to dance^29^. Six male subjects watched 24 dance movements and performed a task unrelated to aesthetics while measuring their brain activity using fMRI. Then they were asked to evaluate each dance stimulus along a set of established aesthetic dimensions after approximately 1 year. The results showed right premotor cortex and bilateral occipital cortices were more active when viewing movements with higher aesthetic ratings compared to movements with low average ratings. Cross, Kirsch, Ticini and Schütz-Bosbach explored the relationship between observers’ aesthetic evaluation of dance and their perceived physical ability to reproduce the movements they watched^30^. They found that occipitotemporal and parietal regions were strongly activated when participants view movements they rated as both aesthetically pleasing and difficult to reproduce. Moreover, inferior parietal lobule, cingulate and supplementary motor areas, ventral premotor cortex, superior temporal sulcus and primary motor cortex were activated when dancers observed and simulated another dancer’s movements^31^.

The research on the aesthetic neural mechanism of dynamic visual stimuli has focused on dance. But we experience many dynamic visual stimuli in our daily life. The neural mechanism of the aesthetic judgments of other dynamic stimuli are not known.

Natural landscapes have great aesthetic and appreciation value. Scenic spots are preferred places for people to travel on holidays. Previous studies explored the neural mechanism of aesthetic judgment of static landscapes or scenes. The contrast landscapes versus non-landscapes produced the activity of lingual gyrus and para-hippocampal place area^32^. Highly preferred scenes compared to less preferred scenes produced greater activation in the right parahippocampal cortex and the ventral striatum^33^. Epstein and Kanwisher revealed that parahippocampal place area (PPA), responds selectively and automatically in functional magnetic resonance imaging (fMRI) to passively viewed scenes, but only weakly to single objects and not at all to faces^34^. Furthermore, the activation likelihood estimation analysis on fMRI experiments of visual aesthetic experience of real-word visual scenes indicated clusters of activation in the parahippocampal gyrus, in the place area, retrosplenial cortex and middle temporal gyrus (MT) in the right hemisphere as well as in the left lingual gyrus^35^.

Natural landscapes can be dynamic, such as a stormy sea, a plunging waterfall, a rippling brook or swaying branches. What are the neural mechanisms that underpin the appreciation of dynamic landscapes? The present study selected natural landscapes which are common and typical of dynamic stimuli in daily life as experimental materials, aiming to explore the neural mechanism underlying aesthetic judgment of dynamic landscapes, and compared the neural mechanisms between the aesthetic judgments of dynamic landscapes and static ones.

Dynamic stimuli typically elicit higher ratings of aesthetic appreciation, and studies manipulating implied motion report effects on aesthetic judgments^36,37^. Even titles of paintings containing words denoting movement can influence the degree of liking for paintings^38^. Based on previous aesthetics studies, we predicted that dynamic landscapes would be judged to be more beautiful than static ones, and would produce stronger activation in MT that mediates visual motion processing, and in emotional processing areas.

## Method

### Ethics statement

The current study was approved by the Institute Ethics Committee, South China Normal University. All experiments were carried out in accordance with relevant guidelines and regulations for human participants laid down by the Institute Ethics Committee. All participants provided written informed consent prior to their participation in the experiments.

### Participants

Twenty-two healthy right-handed university students (11 males) took part in the experiment (age 21.68 ± 2.01). All participants had normal or corrected-to-normal vision, and none of them had a history of neurological or psychiatric disorders. Before participating in the experiment, participants were asked to answer a question about whether they had special experience in painting or art theory or not, and all of them reported that they had no relevant art learning experience.

### Experimental procedures

#### Stimuli

**Figure 1:**
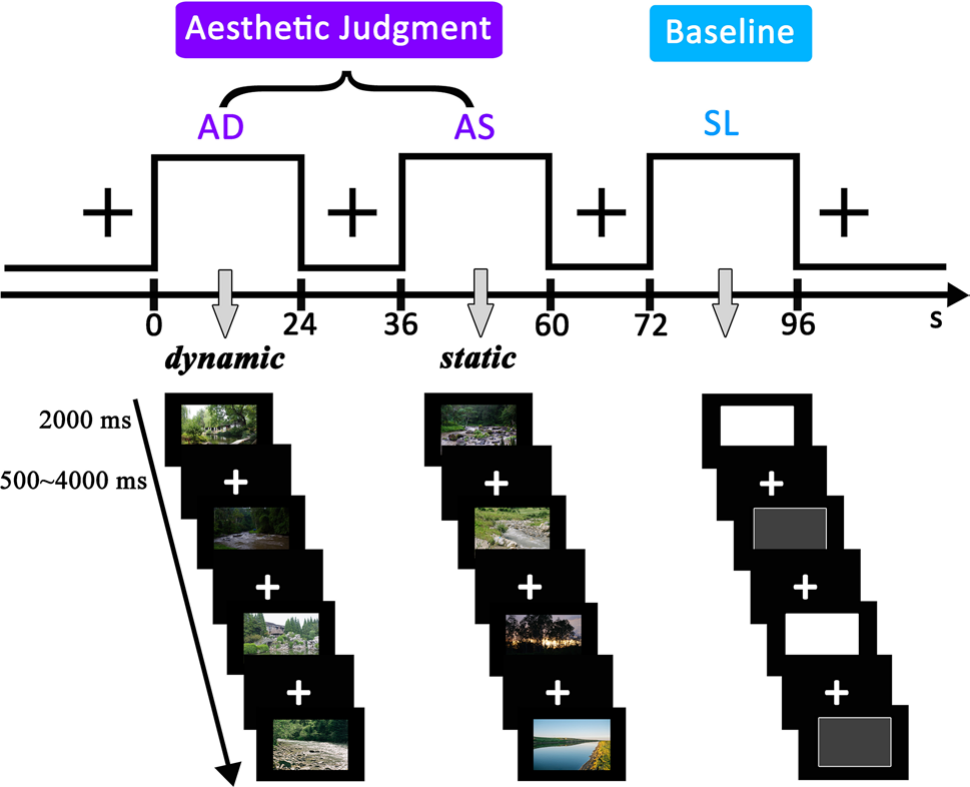
Experimental design, procedure and examples of stimuli. Three types of tasks were performed in separate blocks: AD judgments (beautiful dynamic landscapes vs. neutral dynamic landscapes), AS judgments (beautiful static landscapes vs. neutral static landscapes), SL judgments (high luminance vs. low luminance). Examples in the first column are dynamic landscapes. Examples in the second column are static landscapes; Examples in the third column are grey squares, for high and low luminance, respectively. In this figure, we used photographs taken by the authors instead of examples of the stimuli used in the scanning experiment due to licensing restrictions.

We used three stimuli categories in the experiment: dynamic landscapes, static landscapes, and grey squares (see Fig. 1). Dynamic and static landscapes were formed from the same base image so that they would be equated on other features. For landscapes, an original set of 118 dynamic natural scenery pictures (.gif) were selected from the following websites: http://www.asqql.com/html_zuanti/fengjingtpdq/ and https://www.baidu.com/. All images were cropped to fit a 450 × 300 pixels frame. Corresponding static landscape pictures were created in Adobe Photoshop CS6 from the dynamic natural scenery pictures by selecting a single image frame. Dynamic landscape pictures were converted into dynamic videos (.wmv) in Adobe Flash Player, each 3 s in length.

A separate group of participants (n = 20) evaluated 118 landscapes in terms of beauty, complexity, symmetry, familiarity and dynamic degree. Participants only rated beauty, complexity, symmetry and familiarity of static landscapes because the dynamic sceneries and the static sceneries are the same scene. The four evaluations were made on a 7-point scale. For beauty, 1 indicated that the landscape was not at all beautiful, and 7 indicating that the landscape was extremely beautiful. Complexity and symmetry were also rated on a scale from 1 to 7, 1 for very simple or very asymmetric, and 7 for very complicated or very symmetrical. Familiarity was assessed with 1 indicating that the landscape was extremely unfamiliar to the participant, and 7 indicating that the landscape was extremely familiar. Furthermore, they rated degree of dynamism in the landscapes on a 7-point scale, with 1 indicating that movement was extremely weak, and 7 indicating that movement was extremely strong.

We selected 24 beautiful landscapes and 24 neutral landscapes from the original set of 118 landscapes based on rating results. The norming results were as follows. The mean rating of beauty was significantly higher for beautiful landscapes (5.36 ± 0.48) than for neutral landscapes (2.72 ± 0.34), *F*(1,46) = 474.188, *p* < 0.001. The rating results showed no significant differences in complexity (3.78 ± 0.60; 3.49 ± 0.68, for beautiful and neutral landscapes, respectively), *F*(1,46) = 2.541, *p* = 0.118, symmetry (4.21 ± 0.70; 4.30 ± 0.88, for beautiful and neutral landscapes, respectively), *F*(1,46) = 0.151, *p* = 0.699, familiarity (2.58 ± 0.24; 2.51 ± 0.41, for beautiful and neutral landscapes, respectively), *F*(1,46) = 0.558, *p* = 0.459, and dynamism (4.30 ± 1.15; 4.23 ± 1.01, for beautiful and neutral landscapes, respectively), *F*(1,46) = 0.064, *p* = 0.801.

For grey squares, we used 24 high luminance grey squares (RGB = 255, 255, 255) and 24 low luminance grey squares (RGB = 64, 64, 64) taken from the studies by Zhang et al. (2016, 2017).

#### Task

During the scanning, participants performed two kinds of judgments: aesthetic judgment and square luminance judgment. Aesthetic judgments were performed for both the dynamic and static landscapes; participants were instructed to judge whether the landscape was beautiful or not by pressing buttons on magnet compatible response boxes held in different hands. The square luminance judgment task served as a baseline task, similar to the studies by Tsukiura and Cabeza^39^ and Zhang et al.^40,41^. Participants were instructed to judge if the luminance of the square was high or low and indicate their responses in the same way as in the aesthetic judgment task. The aesthetic judgment required participants to make a right-finger response to beautiful dynamic landscapes or beautiful static landscapes and a left-finger response to neutral dynamic landscapes or neutral static landscapes. The square luminance judgment task required participants to make a right-finger response to high luminance squares and a left finger response to high luminance squares.

#### Procedure

The scanning session included three conditions, aesthetic judgment of dynamic landscapes (AD), aesthetic judgment of static landscapes (AS) and square luminance judgment (SL). Each condition included 48 stimuli and was presented with one repetition, resulting in a total of 96 trials. We used a mixed design with 16 blocks for each of the three conditions. Participants underwent two separate scanner runs; each run consisted of 24 blocks. Block order was fixed and counterbalanced across participants. Each block contained 6 trials and lasted for 24 s. There was a 12 s fixation interval between blocks. A fixation cross was presented for 12 s at the beginning of each run. On each trial the stimulus was presented in the center of the screen for 2 s including response time in random order, with a variable jitter times of 500–4000 ms as inter-stimulus interval (see Fig. 1). Before the experiment, participants performed a training session outside of the scanner with some other stimuli, not those used for the fMRI runs.

### Data acquisition

All MRI data were obtained on a 3 T Siemens Trio Tim MR scanner with a 12-channel phased-array head coil at the Magnetic Resonance Imaging Lab, South China Normal University. A gradient echo-planar imaging (EPI) sequence was used with the following parameters: TR = 2000 ms, TE = 30 ms, flip angle = 90^◦^, matrix size = 64 × 64, FOV = 192 mm, slice thickness = 3 mm, inter-slice gap = 1 mm, and 32 axial slices covering the whole brain. In addition, T1-weighted three-dimensional (3D) structural images were acquired by using a MP-RAGE sequence (TR = 1900 ms, TE = 2.52 ms, flip angle = 9^◦^, voxel-size = 1 mm × 1 mm × 1 mm).

### Data analysis

Image pre-processing was performed using DPABI (http://rfmri.org/dpabi, Yan et al.^42^) and analysis was performed using SPM8 (http://www.fil.ion.ucl.ac.uk/spm/). Slice timing and realignment were performed to correct for the acquisition time delay and head motion. The aligned functional images were then co-registered to the high-resolution T1-weighted structural image, normalized to the standard template based on the MNI reference brain, resampled with voxel size of 3 × 3 × 3 mm^3^, and spatially smoothed with an isotropic 6 mm full width-half-maximum (FWHM) Gaussian kernel. Two participants (one male and one female) were excluded in the subsequent analysis as their images had > 2 mm maximum displacement and > 2° rotation.

At the first-level analysis, a general linear model (GLM) was applied to the fMRI time-series in which stimulus onset was modeled as single impulse response functions, and then convolved with SPM8′s canonical haemodynamic response function (HRF). We modeled six regressors of interest: beautiful dynamic landscapes (BD), neutral dynamic landscapes (ND), beautiful static landscapes (BS), neutral static landscapes (NS), high luminance (HL) and low luminance (LL). Six motion parameters estimated during the realignment procedure were included in the model as covariates of no interest. A high-pass filter with a cut-off of 128 s was applied to remove low-frequency noise. At the first level, analyses were performed individually for each participant and contrast images were subsequently entered into a second-level analysis using a random-effects model.

In order to identify cortical networks involved in the aesthetic judgments of dynamic landscapes and static landscapes, we utilized the square luminance judgment as baseline to control for activity in motor brain regions associated with the key responses. At the group level, we first performed the contrasts of “AD > SL”, “BD > HL”, “ND > LL”, “AS > SL”, “BS > HL” and “NS > LL” using the flexible factorial analysis in SPM8. Based on the above contrasts, we then computed a conjunction between the “AD > SL” and “AS > SL”, “BD > HL” and “BS > HL”, “ND > LL” and “NS > LL” using the minimum statistic approach^43^. Moreover, direct comparisons of “AD > AS”, “BD > BS” and “ND > NS” were conducted to investigate differences in neural mechanisms between aesthetic judgments of dynamic landscapes and static landscapes, which is also the main interest of the present study.

## Results

### Behavioral results

**Figure 2:**
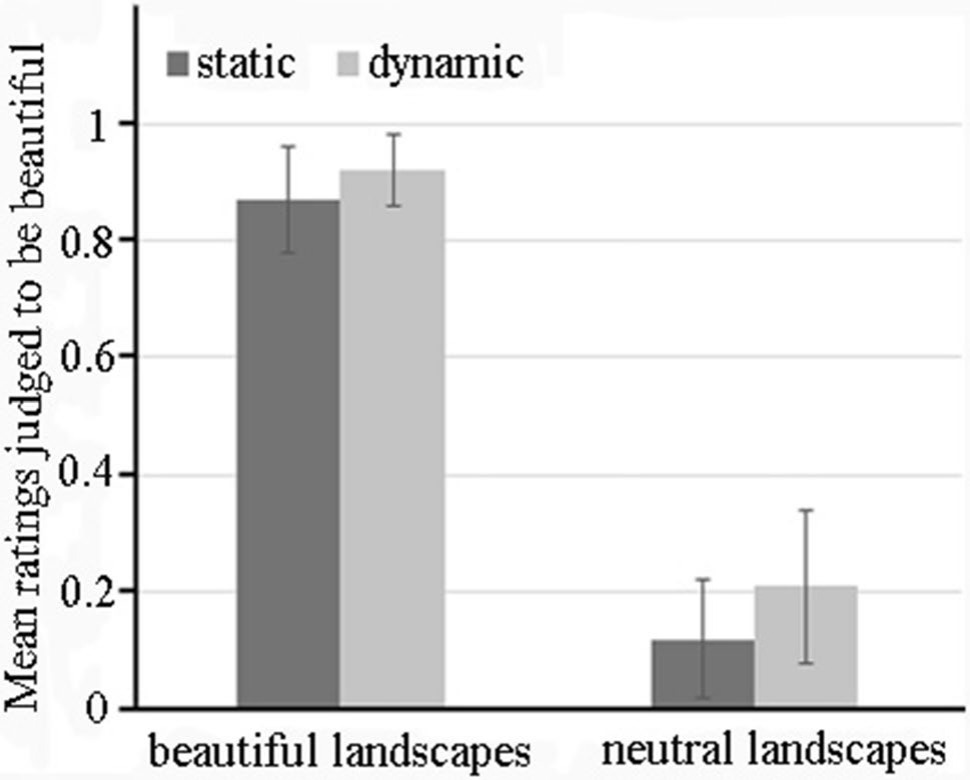
The mean ratings of four types of landscapes judged to be beautiful.

Judgments of beauty, used as a behavioral dependent variable, were calculated as the rates at which dynamic landscapes or static landscapes were judged to be beautiful. For each participant, the two runs were analyzed separately because their judgments may have differed in the two runs. Forty runs were analyzed. For static landscapes, the mean rating was 0.87 ± 0.09 when beautiful, and 0.12 ± 0.10 when neutral. For dynamic landscapes, the mean rating was 0.92 ± 0.06 when beautiful, and 0.21 ± 0.13 when neutral (see Fig. 2).

A 2 (aesthetic qualities: beautiful vs. neutral) × 2 (stimuli types: dynamic landscapes vs. static landscapes) repeated-measures ANOVA revealed a significant main effect of aesthetic qualities, *F* (1, 39) = 1674.28, *p* < 0.001, ƞ^2^ = 0.98. A significant main effect of stimuli types was also found, *F* (1, 39) = 24.18, *p* < 0.001, ƞ^2^ = 0.38, which indicated that the dynamic landscapes were judged to be more beautiful than static landscapes. No significant interaction between aesthetic qualities and stimuli types, *F* (1, 39) = 2.57, *p* = 0.117 were observed.

### fMRI results

#### Brain regions for aesthetic judgments of dynamic landscapes

In order to investigate the neural mechanisms of the aesthetic judgments of dynamic landscapes, the contrast of “AD > SL” was conducted. We found that aesthetic judgments of dynamic landscapes were associated with activity in right fusiform gyrus, left MOG, bilateral IFO, bilateral IFT, left insula, bilateral MCC, left SMA,and right medial superior frontal gyrus (see Table 1). In order to identify brain regions sensitive to aesthetic valence of dynamic landscapes, the “BD > HL” and “ND > LL” contrasts were examined. For the contrast of “BD > HL”, we observed significantly strong activation in the bilateral IOG, bilateral IFO, bilateral IFT, right calcarine, left inferior OFC and bilateral MCC (see Table 1). Similar to the results of the aesthetic judgments of beautiful dynamic landscapes, brain activity related to the contrast of “ND > LL” was also found in large-scale brain networks, including right fusiform gyrus, left middle occipital gyrus, bilateral IFO, bilateral IFT, bilateral insula, bilateral MCC (see Table 1).

**Table 1:** Activated areas correlating with the judgment of dynamic landscapes.

Brain regions	Hemisphere	Peak MNI coordinates			*t-score*	Cluster size
		x	y	z		
**Dynamic landscapes > square luminance**						
Fusiform gyrus	R	30	− 48	− 15	26.49	7919
MOG	L	− 33	− 90	0	26.03	
IFO/ IFT	R	45	12	24	14.00	1403
IFO/ IFT	L	− 45	9	27	12.29	1187
Insula	L	− 33	18	− 3	9.40	
MCC	R/L	3	24	39	10.88	566
SMA	L	− 6	21	63	7.18	
Cerebellum	L	− 33	− 63	− 42	7.78	27
Thalamus	R	6	− 15	0	7.56	32
Medial superior frontal gyrus	R	9	42	51	6.34	20
**Beautiful dynamic landscapes > high luminance**						
IOG	L	− 27	− 93	− 3	18.13	2592
	R	30	− 90	− 3	17.96	2803
IFO/ IFT	R	45	12	24	9.78	486
IFO/ IFT	L	− 42	9	27	8.45	384
Calcarine	R	21	− 54	12	7.08	42
Inferior OFC	L	− 33	33	− 15	6.34	34
MCC	R/L	3	24	39	6.14	61
**Neutral dynamic landscapes > low luminance**						
Fusiform gyrus	R	30	− 48	− 15	19.82	6535
MOG	L	− 33	− 90	0	19.28	
IFT/ IFO	R	48	36	12	10.35	980
Insula	R	33	21	− 3	8.51	
IFT/ IFO	L	− 45	27	12	10.17	729
Insula	L	− 33	18	− 3	7.73	
MCC	R/L	3	24	39	9.25	351

*MOG* Middle occipital gyrus, *IFO* inferior frontal operculum, *IFT* inferior frontal triangle, *MCC* middle cingulate cortex, *SMA* supplementary motor area, *IOG* inferior occipital gyri, *OFC* orbitofrontal cortex.

The activations are FWE corrected at the voxel level and cluster level (*p* < 0.05).

#### Brain regions for aesthetic judgments of static landscapes

To further understand the brain regions involved in aesthetic judgments of static landscapes, we conducted the contrast of “AS > SL”. The results revealed significant activation in right fusiform gyrus, left MOG, bilateral IFO, bilateral IFT, left SMA, left MCC, and right insula (see Table 2). In order to identify brain regions sensitive to aesthetic valence of static landscapes, the “BS > HL” and “NS > LL” contrasts were examined For the contrast of “BS > HL”, we detected significant activation in right IOG, left lingual gyrus, bilateral IFO, bilateral IFT, left SMA and left inferior OFC (see Table 2). The contrast of “NS > LL” revealed significant activity in right fusiform gyrus, left MOG, bilateral IFO, bilateral IFT, bilateral insula, bilateral MCC and left lingual gyrus (see Table 2).

**Table 2:** Activated areas correlating with the judgment of static landscapes.

Brain regions	Hemisphere	Peak MNI coordinates			*t-score*	Cluster size
		x	y	z		
**Static landscapes > square luminance**						
Fusiform gyrus	R	30	− 48	− 15	25.00	3351
MOG	L	− 33	− 90	0	23.99	3111
IFO/IFT	R	45	12	24	12.31	767
IFO/IFT	L	− 45	6	27	10.56	844
SMA	L	0	21	45	8.88	286
MCC	L	− 6	27	33	7.68	
Insula	R	33	21	− 3	7.97	179
**Beautiful static landscapes > high luminance**						
IOG	R	30	− 87	− 6	17.50	2464
Lingual gyrus	L	− 21	− 90	− 15	16.99	2251
IFO/IFT	R	45	12	24	8.63	349
IFO/IFT	L	− 45	6	27	7.59	205
SMA	L	0	21	45	6.13	43
Inferior OFC	L	− 42	45	− 9	5.48	20
**Neutral static landscapes > low luminance**						
Fusiform gyrus	R	30	− 48	− 15	18.37	2618
MOG	L	− 33	− 90	0	17.67	2492
IFO/IFT	R	45	12	21	8.80	446
IFT	L	− 45	30	9	8.67	114
IFO	L	− 42	6	24	7.62	104
Insula	R	33	21	− 3	6.63	26
	L	− 36	15	− 6	6.43	45
MCC	R/L	6	24	36	6.51	120
Lingual gyrus	L	− 18	− 54	3	6.05	29

The activations are FWE corrected at the voxel level and cluster level (*p* < 0.05).

#### Brain regions revealed by conjunction analysis

A Conjunction analysis between “AD > SL” and “AS > SL” were performed to identify common brain activations for aesthetic judgments of dynamic and static landscapes, which showed that the neural networks involved in aesthetic judgments of dynamic landscapes overlapped with those involved in aesthetic judgments of static landscapes (see Table 3). Similarly, the conjunction analysis between “BD > HL” and “BS > HL” revealed that brain regions activated in the aesthetic judgments of beautiful dynamic landscapes covered with those activated in the aesthetic judgments of beautiful static landscapes (see Table 3). Furthermore, conjunction analysis between “ND > LL” and “NS > LL” also showed brain regions of common activation during aesthetic judgments of both neutral dynamic landscapes and neutral static landscapes were basically the areas activated in the aesthetic judgments of neutral static landscapes (see Table 3). In conclusion, the brain regions activated in dynamic landscapes contain the brain regions activated in static landscapes.

**Table 3:** Activation areas in the conjunction analysis between dynamic and static landscapes.

Brain regions	Hemisphere	Peak MNI coordinates			*t-score*	Cluster size
		x	y	z		
**Conjunction of “AD > SL” and “AS > SL”**						
Fusiform gyrus	R	30	− 48	− 15	25.00	3341
MOG	L	− 33	− 90	0	23.99	3092
IFO/IFT	R	45	12	24	12.31	766
IFO/IFT	L	− 45	6	27	10.56	842
SMA	L	0	21	45	8.88	286
MCC	L	− 6	27	33	7.68	
Insula	R	33	21	− 3	7.97	179
**Conjunction of “BD > HL” and “BS > HL”**						
IOG	R	30	− 90	− 6	17.49	2430
Lingual gyrus	L	− 21	− 90	− 15	16.99	2234
IFO/IFT	R	45	12	24	8.63	347
IFO/IFT	L	− 45	6	27	7.59	205
SMA	L	0	21	45	6.08	41
**Conjunction of “ND > LL” and “NS > LL”**						
Fusiform gyrus	R	30	− 48	− 15	18.37	2599
MOG	L	− 33	− 90	0	17.67	2451
IFO/IFT	R	45	12	21	8.80	446
IFT	L	− 45	30	9	8.67	114
IFO	L	− 42	6	24	7.62	104
Insula	R	33	21	− 3	6.63	26
	L	− 36	15	− 6	6.43	45
MCC	R/L	6	24	36	6.51	120
Lingual gyrus	L	− 18	− 54	3	6.05	29

The activations are FWE corrected at the voxel level and cluster level (*p* < 0.05).

#### Cortical differentiation between dynamic and static landscapes

**Figure 3:**
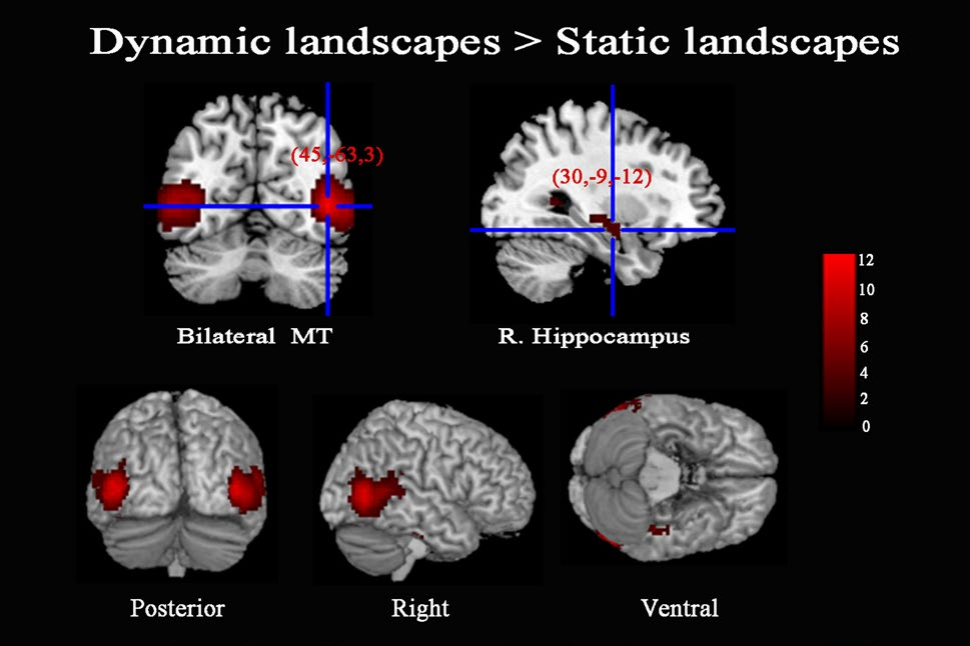
Cerebral areas active in the contrast of “Dynamic landscapes > Static landscapes”.

In order to investigate the difference of the neural mechanism between the aesthetic judgments of dynamic stimuli and static stimuli, we analyzed fMRI activity in the comparisons for dynamic landscapes versus static landscapes. The contrast “AD > AS” revealed significant activation in the bilateral MT, and right hippocampus. No significant activations were found in the contrast of “AS > AD” (see Table 4 and Fig. 3).

**Table 4:** Brain regions of the analysis of variance between dynamic and static landscapes.

Brain regions	Hemisphere	Peak MNI coordinates			*t-score*	Cluster size
		x	y	z		
**Dynamic landscapes > static landscapes**						
MT	R	45	− 63	3	12.72	1081
	L	− 51	− 69	6	10.70	835
Hippocampus	R	30	− 9	− 12	4.41	187
**Static landscapes > dynamic landscapes**						
No significant activations						

*MT* Middle temporal gyrus.

The statistical significance refers to *p* < 0.001 at voxel level (uncorrected), *p* < 0.05 at cluster level (FWE corrected).

To further understand how the differences between dynamic and static landscapes are represented in beauty, a direct comparison was conducted between BD and BS. Results show that stronger activations for beautiful dynamic landscapes, which revealed bilateral MT were significantly activated in the contrast of “BD > BS” (see Table 5). However, there were no significant activations in the contrast of “BS > BD” and “NS > ND”. Meanwhile, stronger activations related to the contrast of “ND > NS” were observed in the right MT, left MOG, bilateral thalamus (see Table 5).

**Table 5:** Brain regions of the analysis of variance between dynamic and static landscapes.

Brain regions	Hemisphere	Peak MNI coordinates			*t-score*	Cluster size
		x	y	z		
**BD > BS**						
MT	R	45	− 60	0	9.18	729
	L	− 51	− 69	9	7.33	463
**BS > BD** No significant activations						
**ND > NS**						
MT	R	45	− 66	3	9.21	716
MOG	L	− 51	− 69	0	8.49	463
Thalamus	R	12	− 6	0	4.44	65
	L	− 21	− 18	6	4.08	91
**NS > ND** No significant activations						

The statistical significance refers to *p* < 0.001 at voxel level (uncorrected), *p* < 0.05 at cluster level (FWE corrected).

## Discussion

The present study investigated differences in neural mechanisms underlying aesthetic judgment of dynamic and static landscapes. As predicted, the behavioral data indicated that dynamic landscapes are judged to be more beautiful than static ones even when images are matched for symmetry, complexity, and familiarity.

Neuroimaging results revealed that regions associated with visual (fusiform gyrus, middle occipital gyrus, et al.), cognitive (inferior frontal gyrus), emotional and reward (insula, orbitofrontal cortex, et al.) processing were commonly activated both in aesthetic judgments of dynamic and static landscapes. This result is consistent with the findings of previous aesthetic research on static visual stimuli^9,13,32,44,45^.

Furthermore, the present study found that stronger activation of the bilateral MT was found in the aesthetic judgments of dynamic landscapes than static ones. MT is one of the main brain regions engaged in the perceptual analysis of visual motion^19,22,46,47^. An artistic work may become more appreciative when it activates more cortical areas (visual or otherwise) and more cortical neurons in the brain (mostly visual brain). A painting generally evokes aesthetic experience through activation of areas V1/V2, V3 andV4. If V5/MT, the motion area, can also be activated (truly as in a movie), the aesthetic appraisal of artwork can be enhanced^48^. Zeki and Stutters (2012) asked participants to rate their preference for kinetic dot patterns. The results revealed that patterns of moving dots that were preferred by participants elicited stronger activity in V5/MT than those patterns that were least preferred^23^. TMS applied over V5/MT while viewing the paintings significantly decreased the perceived sense of motion, and also significantly reduced the liking for abstract painting, suggesting V5/MT activity plays a causal role in the appreciation of abstract art^24^. Therefore, we can conclude that the phenomenon that dynamic stimuli were more beautiful than static ones is closely related to the activation of MT.

The result is consistent with the findings of previous research. Prior studies showed that liking of both representational and abstract paintings positively correlated with dynamism perceived in the painting^49,50^. Di Dio revealed that motor cortex areas were activated in the aesthetic judgment of human subjects and nature scenes, indicating that movement perception plays an important role in aesthetic judgments. Especially, nature paintings may evoke aesthetic processes requiring an additional proprioceptive and sensorimotor component, which is based on the viewer’s own experiences, needs and emotions^25^. Moreover, a study by Battaglia, Lisanby, and Freedberg (2012) found that both observation of an action in the painting and imagery of the painting increased corticomotor excitability by using single and paired-pulse transcranial magnetic stimulation, which indicated that there are motor correlates of the relationship between the esthetic quality of a work and the perception of implied movement within it^26^.

The present study also revealed that compared to the aesthetic judgments of static landscapes, dynamic landscapes triggered stronger activation of right hippocampus. The hippocampus can be related to emotional processing^51,52^. Previous studies have found that hippocampus is an important brain area for evaluating aesthetic and pleasant stimuli^16,53^. In the Copenhagen Neuroaesthetics conference, researchers suggested that hippocampus plays an important role in aesthetic judgments, and compared with other stimuli, when participants watch their favorite works of art, intense activities were found in their hippocampus^54^. Rankin even found that the damage of hippocampus could lead to the decline of individual artistic creativity^55^.

Why is dynamic scenery more beautiful than static scenery? Perhaps it is not just because more brain regions were activated in the aesthetic judgments of dynamic landscapes, but also because dynamic landscapes are more embodied. It has been proposed that a crucial element of aesthetic judgments of artworks consists of the activation of the embodied simulation of actions, emotions, and corporeal sensations, and that these mechanisms are universal^56^. Even static stimuli with a high dynamic content might induce the stimulation of a movement in the observer^7,57^. This embodied experience or this embodied simulation plays an important role in enhancing the aesthetic evaluation of art works through a mechanism of personal involvement in the work itself^49,56,58,59^. Aesthetic preference would be enhanced when action priming is congruent with the artist’s painting style^60^. Ardizzi et al. asked participants to contract the Corrugator Supercilii facial muscles or to refrain from any voluntary facial movement while judging the aesthetic value of painful and neutral facial expressions. The results revealed that participants’ motor enactment of painful facial expressions increased the aesthetic rating of pictorial facial expressions of pain^61^. Dynamic landscapes might trigger greater engagement than static ones. For many positive experiences, greater engagement with and attention to the experience may increase enjoyment^62–64^. This greater engagement made dynamic landscapes more beautiful.

Complexity plays a very important role in aesthetic judgments^14,65,66^. Aitken found, the scores of pleasure and interest both increased with the increase of complexity^67^. Landwehr, Labroo and Hermann manipulated the typicality and complexity of the pictures. The results found that typical and complex cars had a positive impact on car sales^68^. Complex cars are more loved by people. Hence, one of the reasons why dynamic landscapes are more beautiful than static ones may be the complexity brought by dynamic landscapes.

To summarize, the present study fills the gap of neural mechanisms underlying aesthetic judgment of dynamic landscapes. The results showed that compared to static landscapes, dynamic ones are considered more attractive. The neural networks involved in aesthetic judgments of dynamic landscapes overlapped with those involved in aesthetic judgments of static landscapes. Neural correlates of aesthetic appraisal of the two sets of stimuli suggest that judgments of beauty for dynamic landscapes and static ones rely on common neural pathways supporting visual, cognitive, emotional processing. Moreover, visual motion related areas, emotional related areas were significantly activated in the aesthetic judgments of dynamic landscapes than static ones, implying stronger aesthetic experience were elicited by the dynamic landscapes.
